# Comparison between the Chief Care Manager and the Normal Care Manager on Hospitalization and Discharge Coordination Activities in Japan: An Online Cross-Sectional Study of Care Managers in Aichi Prefecture

**DOI:** 10.3390/ijerph191912122

**Published:** 2022-09-25

**Authors:** Yuko Goto, Hisayuki Miura, Naomi Ito

**Affiliations:** Department of Home Care and Regional Liaison Promotion, National Center for Geriatrics and Gerontology, Obu 474-0031, Japan

**Keywords:** care manager, chief care manager, care management, discharge management, hospitalization management, information sharing

## Abstract

The Japanese long-term care insurance system came into operation in the year 2000 and the chief care manager certification system was established in 2006 to improve the quality of care management. Certified chief care managers are expected to perform the role of a specialist. The purpose of this study was to determine the impact of the chief care manager certificate in coordination with hospitals. In this online cross-sectional survey, responses were obtained from 448 care managers engaged in long-term care activities with all in-home long-term care support providers. Among these 448 care managers, 301 had the chief care manager certificate. Of these care managers, ≥90% regularly asked their patients about their “values” and ≥80% provided their patients with hospitalization and discharge support. Of the care managers who provided their patients with hospitalization support, 80% provided the hospitals with information regarding patient “values” at the time of hospitalization, and 50% provided the hospitals with information regarding patient “values” and information. The chief care manager certificate had positive effects on confidence in hospitalization and discharge support. However, no significant difference was observed between the activities of chief and normal care managers in terms of hospitalization and discharge support.

## 1. Introduction

With the increase in the older adult population, the long-term care insurance system came into operation in Japan in the year 2000 to reduce the burden of long-term care on family members. As a result, long-term care insurance services have become available to all older adults. Currently, approximately 20% of older adults use long-term care insurance services in Japan [1,2]. Care managers assigned to older adults who use long-term care insurance services develop care plans and assist them with their daily living and medical needs [3].

Among older adults in Japan, there is an approximately 10-year difference between the healthy life expectancy and the life expectancy at birth [4], and many older adults use long-term care insurance services while receiving treatment for diseases on an inpatient or outpatient basis. Thus, care managers must be able to coordinate with hospital staff members regarding the hospitalization and discharge of older adults as well as develop relationships with the hospital medical professionals to provide the advice and counseling necessary to keep patients’ physical conditions stable during medical treatment. With the increase in the number of older adults requiring long-term care, the Ministry of Health, Labour, and Welfare of Japan established the chief care manager certification system in 2006, advancing the cultivation of human resources capable of ensuring and guiding coordination with more advanced medical institutions, such as hospitals. Care managers and professional caregivers who have studied welfare and long-term care acquire the chief care manager certificate as a career advancement step [5]. However, those who only studied caregiving are less skilled at coordinating with medical professionals and in providing the support required for disease prevention and medical condition management compared to care managers who studied nursing [6].

In addition to their functions as coordinators, chief care managers are also expected to play a supervisory role for other care managers and care staff in the management of older adults; however, studies have shown that chief care managers currently do not fulfill this role fully [7,8].

It has been pointed out that care managers and professional caregivers face challenges when working in close collaboration with hospitals where the vast majority of staff are medical professionals [9]; however, it is unclear how this issue has changed with the introduction of the chief care manager certification system. Moreover, the roles of care managers in coordinating patients’ hospitalizations and discharges with hospitals have not been clearly defined.

Service coordination for the maintenance of users’ daily lives is a characteristic function of average care managers who have studied welfare and long-term care, which otherwise constitutes the background of chief care managers. Distinct from medical professionals in hospitals and other medical institutions, they usually support older adults one-to-one for a long time and may have various information about care recipients, such as their values in the daily lives of older adults, food preferences, relationships with family members, preferred places to receive medical care, and preferred treatment options. Care manager coordination to support decision-making about treatment strategies and locations should therefore be accompanied by careful and timely information sharing with the hospital’s hospitalization and discharge support departments so that older adults can continue to receive medical care with peace of mind [10].

Therefore, for patients to continue living in familiar areas, a project is being implemented wherein the activities of care managers related to patient admission and discharge are supported in the Aichi Prefecture, Japan. Since we were unable to find quantitative survey data regarding the movements and emotions of the care managers in the Aichi Prefecture during patient admission and discharge, the project included a survey to visualize this, the data of which was utilized in the present study.

Most of the preceding studies regarding hospital admission and discharge support have focused on support provided by medical professionals, including home care on hospital admission and discharge support as hospital support. Therefore, most of these studies have set the duration of hospitalization, readmission rate after discharge, post-discharge mortality rate, and medical costs as intervention evaluation items [11,12,13,14,15,16]. Conversely, the effects of early post-discharge nursing home care, rehabilitation care, and social support interventions have also been studied [17,18,19]. Care managers who support the lives of older people in Japan are independent of medical institutions, and distinct from that overseas, many tend to have received welfare education [9].

We were unable to confirm a quantitative survey that visualized the activities and feelings regarding cooperation with hospitals from the perspective of care managers, which can be highly unique to Japan. Furthermore, we were also unable to extract quantitative data visualizing the characteristics of chief care managers who are being trained in Japan.

This study aimed to compare the chief care managers who are expected to perform close coordination functions with hospitals to ordinary care managers as well as to clarify the effects of the chief care manager certificate on the quality of coordination with hospitals.

The involvement of care managers capable of close coordination with hospitals is essential for the lives of older adults in Japan, where the number of older patients continues to rise. Care managers play an important role in judiciously allocating limited medical resources. Thus, this study contributes to the field of medical sociology.

## 2. Materials and Methods

### 2.1. Design

This study is an analytical study using existing data from another project. The data used in the present study were not collected by the survey questions designed for this study.

### 2.2. Participants

The participants were care managers engaged in long-term care activities working with all in-home long-term care support providers who were members of or registered with AICHIKEN KYOTAKU KAIGO SHIEN JIGYOSYA RENRAKU KYOUGIKAI GENERAL INC.ASSOCIATION [AIKAIREN]. The “JIGYOSYA” refer to organizations/companies of provider, and “care managers” refer to individuals, respectively.

The AIKAIREN is an organization that provides education and disseminates information to improve the quality of the care managers, and only providers to which care managers in Aichi Prefecture belong participate in the study. This survey was conducted as part of the hospitalization/discharge coordination support program sponsored by Aichi Prefecture. Therefore, it was best to target care managers who belong to AIKAIREN providers.

The secretariat of AIKAIREN sent the survey manual and participation request in the online survey via e-mail to all members or registered providers. The providers sent survey forms to member care managers, who voluntarily completed the survey using the form.

This survey aimed to clarify the association between the chief care manager certification status and hospitalization/discharge coordination activities with hospitals. Thus, respondents were divided into two groups during logistic regression analyses based on whether or not they had obtained the chief care manager certificate. Assuming that three variables (the age group, number of years of experience and licensed/nonlicensed-nurse status), 3 × 10 = 30/group was the minimum required sample size for the smaller group, with and without the chief care manager certificate [20].

As of 2021, approximately 350 providers belonged to AIKAIREN, implying that an adequate number of participants could be obtained. The AIKAIREN providers were unable to grasp how many care managers belonged to 350 providers and had no statistical data.

### 2.3. Data Collection

NEO MARKETING INC. (Tokyo, Japan) prepared the online survey form, responded to inquiries from participants, and compiled response data. The survey was conducted over two months, from 14 January 2022 to 14 March 2022.

The participants were requested to answer questions about hospitalization/discharge coordination activities for their patients during the 3-month period from May 2021 to July 2021. Before answering questions, all participants provided informed consent for the irreversible publication of response data.

This survey was conducted as part of the hospitalization/discharge coordination support program sponsored by Aichi Prefecture. Additionally, this survey was conducted with the strict approval of the Ethics and Conflict of Interest Review Board of the National Center for Geriatrics and Gerontology (No. 1565).

### 2.4. Definition of Terms

#### 2.4.1. Hospitalization Support

Hospitalization support refers to gathering information before hospitalization to be provided to the hospital, which includes “the submission of information provision forms on hospitalization” to the hospital after hospitalization without delay and activities to contact the parties involved in hospitalization.

In Japan, care managers can be proactively involved in hospitalization support. In addition, hospitalization and discharge support are independently provided.

#### 2.4.2. Discharge Support

Discharge support includes participation in predischarge conferences for a smooth discharge of the user/resident while the user/resident is hospitalized; consultation and contact with community care and home medical care providers; hospital visits to understand the user/resident’s condition; and contacting the user/resident’s family members.

In Japan, hospital discharge support is led by medical institutions, thereby often making it difficult for care managers to be proactively involved in this process. Therefore, for care managers, hospitalization and discharge support are not a series of support activities but independent of each other.

### 2.5. Explanation of Survey Items

There were 29 questions (Table 1). The questions and answer options were refined to minimize erroneous responses via a pre-test conducted by three representatives of AIKAIREN.

#### 2.5.1. Key Statistics

The following information was collected via multiple-choice questions: years of experience as a care manager, professional background (with or without a nursing license), the age group, and chief care manager certification (with or without).

#### 2.5.2. Hospitalization/Discharge Support Activities

The following information was collected via multiple-choice questions: whether the respondent understood the “values,” “desired medical care,” and “goals in life” of his/her patients through daily interactions; the number of patients for whom the respondent provided hospitalization/discharge coordination activities during hospitalization/discharge; and whether the respondent provided patient information to or collected patient information from hospital staff during hospitalization/discharge. In addition, the participants were also asked about the number of information collection activities—e.g., participation in the discharge conference, holding in discussion for long-term care plan—they performed during hospitalization/discharge and whether they provided patient information to the hospital staff. Discussing this information with the patient on a daily basis and supporting the patient’s medical treatment and care based on the information leads to supporting how the patient wants to live and practicing patient-centered care. Furthermore, it is important for hospitals to share these data with hospitals when they need inpatient medical care, as it can provide medical care based on the patient values [21].

#### 2.5.3. Psychological Information

The participants were asked about the anxiety and confidence in coordination activities for their patients during hospitalization/discharge and were requested to answer the questions on a six-point Likert scale, ranging from “very much” to “not at all.”

### 2.6. Statistical Analyses

All respondents were divided into two groups based on whether or not they had obtained the chief care manager certificate, and the chi-square test was used to analyze the associations of the chief care manager certification status with the years of experience, age group, and nursing license (with/without).

The number of care managers who provided hospitalization support was analyzed for those who answered ≥1 to Q7 (How many patients were involved in your hospitalization support during the 3 months from May 2021 to July 2021?).

In terms of the number of times contacted about “predischarge conferences” held by hospitals, care managers who responded ≥1 to Q12 (How many times were you contacted by the hospital about holding a predischarge conference for the patients involved in discharge support during the 3 months from May 2021 to July 2021?) were included in the analyses.

The number of patients for whom care managers provided hospitalization support and the number of patients for whom care managers provided discharge support were summarized separately for care managers with/without the chief care manager certificate and with/without the nursing license. Depending on whether they had a chief care manager certificate or a nursing license, the Mann–Whitney U test was used to evaluate the difference between the number of times hospitalization/discharge support was provided and the number of times care managers had been contacted by hospital about holding a “predischarge conference.” In terms of the evaluation of the difference in the number of times in key statistics of participants’ characteristics, we evaluated the number of people, including care managers, who did not provide support or were not contacted by hospitals.

Next, the following correlations were evaluated: possession of the chief care manager certificate and nursing license; understanding/nonunderstanding of patients’ “values,” “desired medical care,” and “goals in life,” and the experience of being involved in hospitalization support.

We evaluated the correlations between these variables and the number of care managers who responded ≥1 to Q12 (How many times were you contacted by the hospital about holding a predischarge conference for the patients involved in discharge support during the 3 months from May 2021 to July 2021?).

Finally, logistic regression analyses were performed with the certified/noncertified chief care manager status as an independent variable after adjusting for licensed/non-licensed-nurse status and the number of years of experience to assess the effects on the understanding/nonunderstanding of patients’ “values,” “desired medical care,” and “goals in life,” provision/non-provision of information to hospitals at the time of hospitalization, and anxiety/confidence levels in hospitalization/discharge coordination as dependent variables. The anxiety/confidence levels in hospitalization/discharge coordination were converted to binary variables (“yes/no”) before analyses.

Care managers who answered ≥1 to Q7 (How many patients were involved in your hospitalization support during the 3 months from May 2021 to July 2021?) were replaced with “1,” while those who answered 0 were replaced with “0” for these analyses. Similarly, care managers who answered ≥1 to Q12 (How many times were you contacted by the hospital about holding a predischarge conference for the patients involved in discharge support during the 3 months from May 2021 to July 2021.) were replaced with “1,” while those who answered 0 were replaced with “0” for these analyses.

Anxiety was divided into two groups on a 6-point Likert scale: the “anxious” group of respondents who answered “very anxious,” “anxious,” and “moderately anxious,” and the “not anxious” group of respondents who answered “moderately not anxious,” “not anxious,” and “not anxious at all.”

Confidence was divided into two groups on a 6-point Likert scale: the “confident” group of respondents who answered “very confident,” “confident,” “moderately confident,” and the “not confident” group of respondents who answered “moderately not confident,” “not confident,” “not confident at all.”

Data on anxiety about hospitalization support were collected from all respondents in the initial survey, but in this study, we re-analyzed data only for those who answered the question of confidence level in hospitalization support among care managers who answered ≥1 to Q7 (How many patients were involved in your hospitalization support during the 3 months from May 2021 to July 2021?).

This survey was anonymous, and the respondent’s affiliation information could not be grasped at all. Therefore, it was impossible to identify care manages belonging to the same affiliation and adjust them in consideration of their influence. In addition, there were no responses that included missing values in this survey.

The significance level was set to 0.05. SPSS version 28 (IBM SPSS Japan, Tokyo, Japan) was used for data analyses.

### 2.7. Survey Method and Ethical Considerations

IRB Approval Code and Name of the Institution: Approval code: no. 1565 (1 December 2022) from the National Center for Geriatrics and Gerontology.

## 3. Results

### 3.1. Participants’ Characteristics

Responses were obtained from 448 care managers, including 301 who had the chief care manager certificate (67.2%).

Many care managers had gathered information about their patients’ “values,” “desired medical care,” and “goals in life” through daily interactions and were involved in their patients’ hospitalization and discharge (Table 2).

The chief care managers were older, more experienced, more likely to have a nursing license, and more confident in hospitalization/discharge support activities compared to non-chief care managers (Table 3).

Furthermore, the chief care managers did not experience anxiety during hospitalization support activities compared to non-chief care managers (Table 3).

### 3.2. Survey Results

The number of times care managers had been contacted by a hospital about holding a “predischarge conference” and the number of patients for whom care managers provided hospitalization support were summarized separately for care managers with/without the chief care manager certificate, as well as those with/without a nursing license.

The number of care managers who provided hospitalization support was analyzed for 336 care managers who answered ≥1 to Q7 (How many patients were involved in your hospitalization support during the 3 months from May 2021 to July 2021?).

In terms of the number of times contacted about “predischarge conferences” held by hospitals, 217 care managers who answered ≥1 to Q12 (How many times were you contacted by the hospital about holding a predischarge conference for the patients involved in discharge support during the 3 months from May 2021 to July 2021?) were included in the analysis.

The median numbers of patients for who care managers with or without the chief care manager certificate provided hospitalization support were 2. The median value of the “number of times contacted for predischarge conferences” held by hospitals was 1.5 for chief care manager and 1 for those without a chief care manager certificate. (Table 4).

The median numbers of patients for whom care managers with or without the nursing license provided hospitalization support were 2. In addition, the median number of times that care managers with or without a nursing license were contacted for a “predischarge conference” held by medical institutions was 1 (Table 4)

The median value of the “number of times contacted for predischarge conferences” held by hospitals with or without a nursing license was 1 (Table 4).

The difference in the number of times was evaluated by the number of those, including care managers, who responded that they did not provide support or that they had not been contacted.

The Mann–Whitney U test was performed on the number of times hospitalization support was provided, and no significant differences were observed when the groups were divided by the presence or absence of chief care manager certificates (U = 20,043.5, *p* = 0.099). Next, the number of times hospitalization support was provided by groups categorized according to the provision of a nursing license was analyzed using the Mann–Whitney U test; however, no significant difference was observed (U = 14,312.0, *p* = 0.908).

In addition, a significant difference was observed when the number of times discharge support was provided by groups categorized according to the provision of a chief care manager certificate was analyzed via the Mann–Whitney U test (U = 19,358.5, *p* = 0.026). However, when the number of times discharge support was provided was analyzed according to the presence or absence of a nursing license, no significant difference was observed (U = 13,192.0, *p* = 0.218).

In addition, regarding the number of times care managers were contacted about “predischarge conferences” held by hospitals, a significant difference was observed when the Mann–Whitney U test was conducted by dividing the groups according to the presence or absence of a chief care manager certificate (U = 19,503.0, *p* = 0.027). Next, in terms of the number of times they were contacted about “predischarge conferences” held by hospitals, no significant difference was observed when the Mann–Whitney U test was performed by dividing the groups according to the presence or absence of a nursing license (U = 14,161.5, *p* = 0.779).

The Spearman’s correlation analysis revealed no correlations between the chief care manager certificate and the collection of information about patients’ “values,” “desired medical care,” and “goals in life” through daily interactions (r = 0.091, *p* = 0.055).

The Spearman’s correlation analysis revealed no correlations between the nursing license and the collection of information about patients’ “values,” “desired medical care,” and “goals in life” through daily interactions (r = 0.064, *p* = 0.179).

Next, a significant difference was found in the correlation between the number of times care managers who answered that they provided hospitalization support for one or more patients and the chief care manager certificate, but the correlation coefficient was so small that it could be said that there was no correlation (r = 0.121, *p* = 0.026). In addition, there was no correlation between the nursing license and care managers who provided support for one or more hospitalizations (r = −0.055, *p* = 0.311).

Furthermore, no significant association was found between the number of times contacted about the “predischarge conference” held by hospitals and the chief care manager certificate (r = 0.06, *p* = 0.381) or nursing license (r = −0.001, *p* = 0.989) (Table 5).

Next, using logistic regression analysis, nursing license status and years of experience were used as adjustment variables, and chief care manager certification status was used as an independent variable. Care managers who answered ≥1 to Q7 (How many patients were involved in your hospitalization support during the 3 months from May 2021 to July 2021?) were replaced with “1,” while those who answered 0 were replaced with “0” for these analyses. Similarly, care managers who answered ≥1 to Q12 (How many times were you contacted by the hospital about holding a predischarge conference for the patients involved in discharge support during the 3 months from May 2021 to July 2021?) were replaced with “1,” while those who answered 0 were replaced with “0” for these analyses.

We analyzed the associations between the chief care manager certification status and the collection of information about patients’ “values,” “desired medical care,” and “goals in life” through daily interactions and involvement in hospitalization/discharge, but it was not significant. (Table 6).

Compared with normal care managers, a higher proportion of chief care managers had nursing licenses and tended to have more years of experience. Thus, adjustments were made using these variables. It was confirmed that senior chief care managers tended to be older than normal care managers; however, this was considered strongly linked to years of experience, and hence, age was not adjusted. No influence of a chief care manager certificate was observed on the activities used to collect information on patients’ “values,” “desired medical care,” and “goals in life” on a daily basis and the activities involved in patient admission and discharge support.

In addition, no effect of the chief care manager certificate was confirmed on providing hospitalization support or being contacted about “predischarge conferences” held by hospitals.

The anxiety/confidence levels in hospitalization/discharge coordination were converted to binary variables (“yes/no”) in advance for analyses as dependent variables.

Furthermore, the analysis of associations between the chief care manager certification status along with the provision of information at least once at hospital hospitalization about patients’ “values,” “desired medical care,” and “goals in life” and confidence/anxiety in hospitalization support revealed a significant positive effect only between the chief care manager certification status and confidence in hospitalization support (Table 7). Upon using logistic regression analysis, nursing license status and years of experience were used as adjustment variables, and chief care manager certification status was used as an independent variable.

Here again, the chief care managers with a chief care manager certificate showed a higher tendency of having a nursing license than normal care managers, as well as more years of experience. Thus, we used these variables as adjusting factors. In this procedure, no influence of a chief care manager certificate was observed in the activities that provided information regarding patients’ “values,” “desired medical care,” and “goals in life” to hospitals and no anxiety in oneself regarding hospitalization support.

Only on confidence in hospitalization support, the impact of having a chief care manager certificate could be confirmed.

The anxiety/confidence levels in hospitalization/discharge coordination were converted to binary variables (“yes/no”) in advance for analyses as dependent variables.

Anxiety was divided into two groups on a 6-point Likert scale: the “anxious” group of respondents who answered “very anxious,” “anxious,” and “moderately anxious,” and the “not anxious” group of respondents who answered “moderately not anxious,” “not anxious,” and “not anxious at all.”

Confidence was divided into two groups on a 6-point Likert scale: the “confident” group of respondents who answered “very confident,” “confident,” “moderately confident,” and the “not confident” group of respondents who answered “moderately not confident,” “not confident,” “not confident at all.”

Furthermore, the analysis of associations between the chief care manager certification status along with the provision of information at least once at hospital hospitalization about patients’ “values,” “desired medical care,” and “goals in life” and confidence/anxiety in discharge support revealed a significant positive effect only between the chief care manager certification status and confidence in discharge support (Table 8). Upon using logistic regression analysis, nursing license status and years of experience were used as adjustment variables, and chief care manager certification status was used as an independent variable.

Here again, a higher proportion of chief care managers with chief care manager certificate had nursing licenses compared with normal care managers and tended to have more years of experience. Thus, we used these variables as adjusting factors. In this procedure, there was no influence of a chief care manager certificate on the activities providing information on patients’ “values,” “desired medical care,” and “goals in life” to hospitals, the absence of anxiety for discharge support, and information provision about patients’ condition within one month after discharge.

The influence of having a chief care manager certificate was observed only on confidence in discharge support.

## 4. Discussion

To the best of our knowledge, this is the first study on care managers’ coordination activities with hospitals. This study revealed care managers’ daily interactions with patients, their involvement in the hospitalization/discharge of patients, and the effects of the chief care manager certification status and other related factors on care managers’ hospitalization/discharge coordination activities with hospitals.

### 4.1. Care Managers’ Assistance to Patients during Their Hospitalization/Discharge and Related Activities: “Hospitalization/Discharge Support in the Community”

In the present survey, the participants were asked about their activities between May 2021 and July 2021. The following five points were strongly emphasized in the 2021 revision to long-term care fees, and the current changes may reflect the effects of the fee revision.

Point 1: Improving response capacities to infectious diseases and disasters; Point 2: Promoting a community-based integrated care system; Point 3: Promoting independence support/exacerbation prevention efforts; Point 4: Securing human resources and innovate field sites in long-term care; and Point 5: Ensuring the stability/sustainability of the system [22]. In particular, cooperation and coordination between hospital medical care and long-term care, discussion support for end-of-life care, and fee evaluation for improving the care management quality are ongoing to promote the community-based integrated care system (Point 2) and may have influenced care managers’ activities related to the present survey.

The results of this survey showed that ≥90% of care managers collected information about patients’ “values,” “desired medical care,” and “goals in life” through daily interactions and ≥80% of care managers were involved in their patients’ hospitalization/discharge support. Of the care managers who provided their patients with hospitalization support, 80% provided the hospitals with information about patients’ “values,” “desired medical care,” and “goals in life” at hospitalization. On the contrary, only 50% of the care managers who provided their patients with discharge support provided the hospitals with information about patients’ “values,” “desired medical care,” and “goals in life.” Furthermore, only 40% of the participants provided the hospitals with information about their patients’ conditions within one month of discharge.

In addition, it became clear that chief care managers received a significantly higher number of calls for “predischarge conferences” held by hospitals, and that the number of times they were involved in discharge support was significantly higher.

Previous studies have pointed out the difficulty of information sharing between care managers and hospital nurses, who play a central role in in-hospital care [23,24,25]. The results of the present survey suggest that care managers mainly receive information from hospitals. The results of treatments and examinations frequently show that a patient’s condition at discharge differs from that at hospitalization. Thus, there may be differences between the patient information that care managers can provide at hospitalization and the patient information that care managers can provide at discharge.

Previous studies also pointed out that trustworthy and human-centered relationships between patients and care managers are important in the practice of supporting older adults with diseases that require treatment [26,27,28]. Care managers who support patients’ lives on continual basis share information with them about their “values,” “desired medical care,” and “goals in life,” which is important information about patients. In Japan, where many older patients are repeatedly hospitalized and discharged, care managers in such a position are expected to actively provide the relevant information to hospital staff members who interact with patients on a short-term basis, as well as conduct care management in such a way that it facilitates the continuous provision of care in accordance with patients’ values.

### 4.2. Society’s Needs for Care Managers and Chief Care Manager Certification and the Behavioral Change Factors Ofcare Managers

The chief care managers were older, had longer years of experience, had more licensed-nurse status, and were more confident in hospitalization/discharge support activities than non-chief care managers. This study demonstrated that the chief care manager certification status had positive effects only on the confidence during hospitalization/discharge support and had no particular effects on other care management activities.

It was also revealed that chief care managers received significantly more calls for predischarge conferences held by hospitals, and that they were involved in discharge support significantly more times. It is conceivable that this may have led to the confidence of the chief care managers in these procedures. In Japan, in 2018, it became a rule that managers of in-home care support offices (companies where care managers work) must be certified as chief care managers. We have not been able to identify the factors that lead to a significantly higher number of calls from hospitals for chief care managers; however, factors such as the fact that many chief care managers are organizational managers and have the trust of hospitals may have an influence.

Previous studies have shown that anxiety and self-confidence affect self-efficacy [29,30,31], as well as the importance of self-efficacy in promoting behavioral change has been clarified [29,30,31,32,33].

Confidence is an important factor in changing activity [34,35].

However, high self-confidence alone does not benefit patients. It was suggested that the education to obtain the current chief care manager qualification may not have resulted in acquiring the necessary education that changes the activities of care managers.

Although compared with normal care managers, chief care managers received more calls about “predischarge conferences” from hospitals and were more involved in discharge support, no differences between normal care managers and chief care managers were found in providing information on discharge support or providing patient information within one month after discharge.

Chief care managers, as core personnel in the community-based integrated care system, are expected to play a major role in improving the quality of both care management and human resource development [36,37]. Chief care managers are also expected to play a role in the consolidation of cooperation with medical care at hospitalization/discharge; however, the findings of the present study did not clearly show the characteristics of certified chief care managers. Previous studies have also shown concerns and issues associated with the functions of chief care managers [7,8]. The results of the present study can serve as a foundation for the development of improved training programs and quality assessment mechanisms for chief care managers to improve the overall care management quality.

The competency for cooperation with hospitals Is also an important factor of chief care managers. Furthermore, chief care managers are expected to play a central role in multidisciplinary teams. The results of the present survey showed that different percentages of care managers provided hospitals with information about patients’ “values,” “desired medical care,” and “goals in life” at hospitalization versus discharge, suggesting that hospitals expect care managers to play different roles in the two settings. However, active information transmission and team leading by care managers who have a wealth of patient information are required to coordinate patient-centered continuous care. As there is a high expectation for care managers’ roles in patient-centered care coordination and chief care managers’ educational roles in care manager training are also increasing, further improvements in training programs and quality management efforts through quality assessment are required to improve the competence of chief care managers.

### 4.3. Limitations

Given that the survey was conducted online, and all the participants of this survey were care managers who were engaged in long-term care activities in Aichi Prefecture and were either members of or registered with AIKAIREN, it is possible that the responses were skewed (subject bias). The AIKAIREN is an organization that provides training and consultation support services to improve the quality of care managers. Since care managers who wish to receive education and support for skill improvement and quality improvement are actively registered, care managers who can be more motivated to educate and act than general care managers belong to them. A large-scale survey of care managers from various regions and the characterization of chief care managers are necessary to visualize care managers’ activities at hospitalization/discharge. Additionally, unobserved confounders could lead to inconsistencies in the estimates.

The hospitalization/discharge support differs depending on the structure and insurance system of each country. In addition, the status of care managers’ qualifications and the certification of “chief” care managers differ from country to country. In other words, the results of this research could be strongly influenced by the environment unique to Japan, and it may be difficult to apply the findings of this research to other countries. In addition, questions related to confidence and anxiety for hospitalization/discharge support were 6-point Likert scale responses, which may have led to biased responses.

## 5. Conclusions

The Ministry of Health, Labour, and Welfare of Japan had established the chief care manager certification system in 2006, advanced the cultivation of human resources capable of ensuring and guiding coordination with more advanced medical institutions, such as hospitals.

It was clarified that the chief care managers were older, had longer years of experience, had more licensed-nurse status, and were more confident in hospitalization/discharge support activities than nonchief care managers. In addition, it had no particular effects on other care management activities during hospitalization/discharge support.

However, the reason for chief care managers receiving more calls about “predischarge conferences” from hospitals and being more involved in discharge support remains unclarified.

## Figures and Tables

**Table 1:** A part of questions.

No.	Questions	The Survey of Respondent
Q1	Years of experience as a care manager	All respondents ※
Q2	Background licenses	All respondents
Q3	Age group	All respondents ※
Q4	Presence or absence of chief care manager certificate	All respondents
Q5	With or without hearing patient “values,” “desired medical care,” and “goals in life” on a daily basis	All respondents
Q6	With or without having been involved in the hospitalization and discharge support of their patients during the three months from May 2021 to July 2021	All respondents
Q7	How many patients were involved in your hospitalization support during the three months from May 2021 to July 2021	Only respondents of Q6
Q8	Did you provide information to medical institutions on patient “values,” “hope for medical treatment,” and “goals in life,” in the hospitalization support even once during the 3 months from May 2021 to July 2021?	Only respondent(s) (≥1) of Q7
Q9	Degree of your confidence regarding providing hospitalization support	Only respondent(s) (≥1) of Q7 ※
Q10	Degree of your anxiety about providing hospitalization support	Only respondent(s) (≥1) of Q7 ※
Q11	How many patients were involved in your discharge support during the three months from May 2021 to July 2021	All respondents
Q12	How many times had you been contacted by hospital about holding a predischarge conference among the patients involved in discharge support during the three months from May 2021 to July 2021	All respondents
Q13	Degree of your confidence related to providing discharge support	Only respondent(s) (≥1) of Q12 ※
Q14	Degree of anxiety about providing discharge support	Only respondent(s) (≥1) of Q12 ※
Q15	Did you provide information to medical institutions on patient “values,” “hope for medical treatment,” and “goals in life” in the discharge support even once during the 3 months from May 2021 to July 2021?	All respondents
Q16	Had you reported the status of patients to medical institutions within 1 month after the discharge of patients who were involved in your discharge support, during the 3 months from May 2021 to July 2021?	All respondents

※ The Likert-type scale was used for answers.

**Table 2:** Key statistics.

		With Chief Care Manager Certificate		Without Chief Care Manager Certificate		χ^2^-Value	*p*-Value
		N	%	N	%		
		301	67.2	147	32.8		
Age group	(*n* = 448)	301	67.2	147	32.8	39.581	<0.001 *
	20–29 years	0	0	1	100		
	30–39 years	4	19.0	17	81.0		
	40–49 years	82	58.2	59	41.8		
	50–59 years	140	74.1	49	25.9		
	60–69 years	70	79.5	18	20.5		
	≥70 years	5	62.5	3	37.5		
Number of years of experience	(*n* = 448)	301	67.2	147	32.8	158.575	<0.001 *
	≤4 years	3	4.5	64	95.5		
	5–9 years	60	61.2	38	38.8		
	10–14 years	108	82.4	23	17.6		
	15–19 years	95	87.2	14	12.8		
	≥20 years	35	81.4	8	18.6		
With a nursing license (*n* = 78) The subjects were care managers who answered that they had a nursing license.		60	76.9	18	23.1	4.060	0.044 *
Number of care managers who collected information about “values,” “desired medical care,” and “goals in life” of their patients through daily interactions (*n* = 422)		288	68.2	134	31.8	3.699	0.054
Number of care managers who were involved in the hospitalization and discharge of their patients (*n* = 360) The subjects were care managers who answered that they were involved in the hospitalization and discharge of patients.		246	68.3	114	31.7	1.091	0.296
Whether or not care managers provided information about patients’ “values,” “desired medical care,” and “goals in life” to hospitals in hospitalization on support at least once (*n* = 284) The subjects were care managers who answered that they provided information about patient’s “values,” “desired medical care,” and “goals in life” to hospitals during hospitalization at least once.		196	69.0	88	31.0	1.772	0.183
Number of care managers who provided information about patients’ “values,” “desired medical care,” and “goals in life” to hospitals in discharge support at least once (*n* = 260) The subjects were care managers who answered that they had provided hospital staff with information on the patient’s “values,” “desired medical care,” and “goals in life” at least once when supporting a patient’s discharge.		176	67.7	84	32.3	0.072	0.789
Number of care managers who provided patient information to hospitals within one month of discharge (*n* = 179) The subjects were care managers who answered that they provided the patient’s information to the hospitals within 1 month of discharge.		128	71.5	51	28.5	2.525	0.112

* *p* < 0.05. Percentages in Table 2 refer to % within rows.

**Table 3:** Degrees of confidence and anxiety in hospitalization/discharge support.

		With a Chief Care Manager Certificate		Without a Chief Care Manager Certificate		χ^2^-Value	*p*-Value
		N	%	N	%		
Confidence level in hospitalization support	(*n* = 336) The subjects were only care managers who answered that they were involved in patients’ hospitalization and discharge support during the 3 months from May 2021 to July 2021.	227		109		18.269	0.001 *
	Very confident	2	0.9	0	0		
	Confident	28	12.3	6	5.5		
	Moderately confident	139	61.2	52	47.7		
	Moderately not confident	58	25.6	50	45.9		
	Not confident	0	0	0	0		
	Not confident at all	0	0	1	0.9		
Anxiety level regarding hospitalization support	(*n* = 336,448)	227		109		13.765	0.017
	Not anxious at all	7	3.1	2	1.8		
	Not anxious	27	11.9	7	6.4		
	Moderately not anxious	85	37.4	28	25.7		
	Moderately anxious	97	42.7	58	53.2		
	Anxious	9	4.0	11	10.1		
	Very anxious	2	0.9	3	2.8		
Confidence level in discharge support	(*n* = 217) The subjects were only care managers who answered that they were contacted by the hospital about holding a predischarge conference for the patients involved in discharge support during the 3 months from May 2021 to July 2021.	156		61		13.552	0.009 *
	Very confident	2	1.3	0	0		
	Confident	14	9.0	1	1.6		
	Moderately confident	90	57.6	27	44.3		
	Moderately not confident	48	30.8	29	47.5		
	Not confident	2	1.3	4	6.6		
	Not confident at all	0	0	0	0		
Anxiety level regarding discharge support	(*n* = 217) The subjects were only care managers who answered that they were contacted by the hospital about holding a predischarge conference for the patients involved in discharge support during the 3 months from May 2021 to July 2021.	156		61		7.121	0.13
	Not anxious at all	3	1.9	0	0		
	Not anxious	17	10.9	2	3.3		
	Moderately not anxious	56	35.9	20	32.8		
	Moderately anxious	72	46.2	32	52.5		
	Anxious	8	5.1	7	11.4		
	Very anxious	0	0	0	0		

* *p* < 0.05. Percentages in Table 3 refer to % within columns.

**Table 4:** Associations between chief care manager certification status and nursing license status of care managers and the number of patients for whom they provided hospitalization/discharge support.

	**Number of patients for whom care managers provided** **hospitalization support** **(*n* = 336)** The subjects were care managers who answered ≥1 to Q7 (How many patients were involved in your hospitalization support during the 3 months from May 2021 to July 2021?).						**Number of times contacted about “predischarge conferences” held by hospitals** **(*n*=2174)** The subjects were care managers who answered ≥1 to Q12 (How many times had you been contacted by the hospital about holding a predischarge conference for the patients involved in discharge support during the 3 months from May 2021 to July 2021?).					
	***n***	**Median**	**Minimum**	**Maximum**	**SD** **※ 1**	**IQR** **※ 2**	***n***	**Median**	**Minimum**	**Maximum**	**SD** **※ 1**	**IQR** **※ 2**
With chief care manager certificate	226	2	1	12	2.013	3	156	1.5	1	17	2.037	1
Without chief care manager certificate	106	2	1	10	2.061	2	61	1	10	10	1.707	1
	**Number of patients for whom care managers provided hospitalization support** **(*n* = 336)** The subjects were care managers who answered ≥1 to Q7 (How many patients were involved in your hospitalization support during the 3 months from May 2021 to July 2021?).						**Number of times contacted about “predischarge conferences” held by hospitals** **(*n* = 217)** The subjects were care managers who answered ≥1 to Q12 (How many times were you contacted by the hospital about holding a predischarge conference for the patients involved in discharge support during the 3 months from May 2021 to July 2021?).					
	***n***	**Median**	**Minimum**	**Maximum**	**SD** **※ 1**	**IQR** **※ 2**	***n***	**Median**	**Minimum**	**Maximum**	**SD** **※ 1**	**IQR** **※ 2**
With a nursing license	61	2	1	7	1.588	3	39	1	10	10	1.709	2
Without a nursing license	275	2	1	12	2.114	3	178	1	1	17	1.999	1

※1 standard deviation; ※2 interquartile range.

**Table 5:** Association between chief care manager certification status and nursing license status of care managers and the number of care managers who collected information about “values,” “desired medical care,” and “goals in life” of their patients through daily interactions, the number of care managers who were involved in the hospitalization of their patient, and the number of times contacted about the “predischarge conferences” held by hospitals.

	**Number of care managers who collected information about “values,” “desired medical care,” and “goals in life” of their patients through daily interactions** **(*n* = 422)** The subjects were care managers who answered that they collected information about “values,” “desired medical care,” and “goals in life” of their patients through daily interactions.		**Number ofpatients for whom care managers provided hospitalization support** **(*n* = 336)** The subjects were care managers who answered ≥1 to Q7 (How many patients were involved in your hospitalization support during the 3 months from May 2021 to July 2021?).		**Number of times contacted about the “predischarge conferences” held by hospitals** **(*n* = 217)** The subjects were care managers who answered ≥1 to Q12 (How many times had you been contacted by hospital about holding a predischarge conference for the patients involved in discharge support during the 3 months from May 2021 to July 2021?).	
	**r** **※ 1**	***p*-Value**	**r** **※ 1**	***p*-Value**	**r** **※ 1**	***p*-Value**
With/without chief care manager certificate	0.091	0.055	0.121	0.026 *	0.06	0.381
With/without nursing license	0.064	0.179	−0.055	0.311	−0.001	0.989

※1 Correlation coefficient; * *p* < 0.05.

**Table 6:** Associations between the chief care manager certification status with the daily collection of information from patients, involvement in hospitalization and being contacted about “predischarge conferences” held by hospitals.

	**Collection of information about patients’ “values,” “desired medical care,” and “goals in life” through daily interactions** **(*n* = 422)** The subjects were care managers who answered that they collected information about “values,” “desired medical care,” and “goals in life” of their patients through daily interactions.				**Involvement in hospitalization** **(*n* = 336)** The subjects were care managers who answered ≥1 to Q7 (How many patients were involved in your hospitalization support during the 3 months from May 2021 to July 2021?).				**Number of times contacted about “predischarge conferences” held by hospitals** **(*n*= 2174)** The subjects were care managers who answered ≥1 to Q12 (How many times were you contacted by hospital about holding a predischarge conference for the patients involved in discharge support during the 3 months from May 2021 to July 2021?).			
	***n***	**OR** **※ 3**	**95% CI** **※ 2**	***p*-Value**	***n***	**OR** **※ 3**	**95% CI** **※ 2**	***p*-Value**	***n***	**OR** **※ 3**	**95% CI** **※ 2**	***p*-Value**
With chief care manager certificate	288	1.444	0.549–3.797	0.456	227	0.519	0.267–1.006	0.052	156	1.320	0.805–2.163	0.271
Without chief care manager certificate	134				109				61			

※ 2 confidence interval; ※ 3 odds ratio. Adjusted variables were licensed/non-licensed-nurse status and the number of years of experience.

**Table 7:** Associations between the chief care manager certification status and hospitalization support practice, confidence, and anxiety.

	**Confidence in hospitalization support** **(*n* = 227)** The subjects were care managers who answered that they were confident in providing hospitalization support among care managers who answered that they were involved in hospitalization support in the 3 months from May 2021 to July 2021.				**No anxiety for hospitalization support** **(*n* = 208)** The subjects were care managers who answered “no anxiety” about hospitalization support among all respondents.				**Care managers provided information about patients’ “values,” “desired medical care,” and “goals in life” to hospitals for hospitalization support at least once** **(*n* = 284)** The subjects were care managers who provided information about patients’ “values,” “desired medical care,” and “goals in life” to hospitals for hospitalization support at least once among care managers who answered that they were involved in hospitalization support during the 3 months from May 2021 to July 2021.			
	***n***	**OR** **※ 3**	**95% CI** **※ 2**	***p*-Value**	***n***	**OR** **※ 3**	**95% CI** **※** **2**	***p*-Value**	***n***	**OR** **※ 3**	**95% CI** **※ 2**	***p*-Value**
With chief care manager certificate	169	2.005	1.162–3.461	0.013 *	154	1.32	0.83–2.098	0.24	196	1.374	0.688–2.747	0.368
Without chief care manager certificate	58				54				88			

* *p* < 0.05; ※ 2 confidence interval; ※ 3 odds ratio. Adjusted variables were licensed/non-licensed-nurse status and the number of years of experience.

**Table 8:** Association between the chief care manager certification status and discharge support practice, confidence, and anxiety.

	**Confidencein discharge support** **(*n* = 134)** The subjects were care managers who answered “confident” in discharge support among care managers who answered that they were contacted by the hospital about holding a predischarge conference for the patients involved in discharge support during the 3 months from May 2021 to July 2021.				**No anxiety in discharge support** **(*n* = 98)** The subjects were care managers who answered “not anxious” in discharge support among care managers who answered that they were contacted by the hospital about holding a predischarge conference for the patients involved in discharge support during the 3 months from May 2021 to July 2021.			
	***n***	**OR** **※3**	**95% CI** **※** **2**	***p*-value**	***n***	**OR** **※** **3**	**95% CI** **※** **2**	***p*-value**
With chief care manager certificate	106	2.268	1.103–4.6665	0.026*	76	1.441	0.705–2.946	0.317
Without chief care manager certificate	28				22			
	**Care managers provided information about patients’ “values,” “desired medical care,” and “goals in life” to hospitals in discharge support at least once** **(*n* = 260)** The subjects were care managers who answered that they provided information about patients’ “values,” “desired medical care,” and “goals in life” to hospitals in discharge support at least once among all respondents.				**Number of care managers who provided patient information to hospitals within one month of discharge** **(*n* = 179)** The subjects were care managers who answered that they provided patient information to hospitals within 1 month of discharge of the patient involved in discharge support during the 3 months from May 2021 to July 2021 among all respondents.			
	***n***	**OR** **※** **3**	**95% CI** **※** **2**	***p*-value**	***n***	**OR** **※** **3**	**95% CI** **※** **2**	***p*-value**
With chief care manager certificate	176	0.946	0.596–1.501	0.812	128	1.167	0.731–1.863	0.511
Without chief care manager certificate	84				51			

* *p* < 0.05; ※ 2 confidence interval; ※ 3 odds ratio. Adjusted variables were licensed/non-licensed-nurse status and the number of years of experience.

## Data Availability

All data generated or analyzed during this study are included in this published article.
